# Treatment Options for Avoidant/Restrictive Food Intake Disorder (ARFID) in Young People with Autism Spectrum Disorder (ASD)

**DOI:** 10.1192/bjo.2026.11149

**Published:** 2026-06-30

**Authors:** Huma Zafar, Khalid Karim

**Affiliations:** Leicester Partnership NHS Trust, Leicester, United Kingdom

## Abstract

**Aims:**

To identify effective treatment approaches for ARFID in children and young people with Autism and to highlight the importance of family-based and multidisciplinary interventions to improve nutritional intake, mealtime behaviours, improve quality of life for children and caregivers and reduce long-term clinical burden through early intervention.

**Methods:**

Review of existing literature on ARFID and Autism Spectrum Disorder. Sources included Systematic review, Treatment protocols, Clinical trials and Peer review journals.

**Results:**

Evidence suggests there is high prevalence of ARFID in children with ASD. Parental acceptability and responsibility remains the core and significant area in the treatment journey.

Family based Treatment (FBT-ARFID) is very much effective and is the leading evidence-based manualised treatment for children that are of school-going age and adolescents. It helps the chosen family to re-establish healthy eating in their loved ones and also reduce the symptoms of the disorder–parents take lead in helping children recover with the help of multi-disciplinary approaches.

Family-based treatments and Unified protocol programmes (combination of FBT and CBT) to manage anxiety, food rigidity and sensory sensitivities, make mealtimes less stressful and help with an increase in the oral intake. Many studies have shown that children undergoing FBT have shown progressive gain in weight and also have reduced symptoms,giving parents and family hope in re-feeding their child.

Many school-going children and young adults can also have benefits from individual Cognitive behavioural therapy (CBT). CBT helps people change their thinking patterns and behaviours to cope with changes in mood and help them cope overall.

**Conclusion:**

ARFID and ASD co-occur due to shared sensory, cognitive rigidity and behavioural traits.

Early, individualised treatment can prevent severe nutritional and medical complications. Family-based interventions including family-based therapy and CBT are key to successful outcomes.

Investing in a structured ARFID-Autism service represents a high-impact, cost-effective solution. Combination of Family basedtreatment, early screening, and multi-disciplinary approaches can offer improved clinical outcomes for children and families.

